# Quantifying generational and geographical inequality of climate change

**DOI:** 10.1038/s41598-023-35690-8

**Published:** 2023-05-25

**Authors:** Emma Hadré, Jonas Küpper, Janina Tschirschwitz, Melissa Mengert, Inga Labuhn

**Affiliations:** Institute of Geography, University of Bremen, Celsiusstr. 2, 28359 Bremen, Germany

**Keywords:** Climate sciences, Environmental social sciences

## Abstract

We relate greenhouse gas emissions and global warming experienced over a lifetime by individual birth cohorts, resolved by world regions. We reveal outstanding geographical inequality between high- and low-emission regions corresponding to the nations of the Global North and Global South, respectively. Additionally, we highlight the inequality different birth cohorts (generations) experience regarding the burden of recent and ongoing warming temperatures as a time-delayed consequence of past emissions. We achieve precise quantification of the number of birth cohorts and populations who see a difference between Shared Socioeconomic Pathways (SSPs), emphasizing the potential for action and the chances for improvement that exist under the different scenarios. The method is designed to realistically display inequality, as it is experienced by people while motivating action and change needed to achieve emission reduction to reduce climate change and generational and geographical inequality simultaneously.

## Introduction

The global mean surface temperature has increased by more than 1 °C relative to the preindustrial period^1,2^. This global warming is caused by anthropogenic emissions of greenhouse gases (GHG), most importantly CO_2_^1^. The excess energy accumulated in the earth system is changing the global climate, causing glacier and ice cap melt, rising sea levels, increasing frequency and severity of weather extremes such as heatwaves, and increasing heat-related mortality rates^2^. Of all populations, children are especially vulnerable to the effects of climate change^3,4^**.**

The effects of climate change are not spatially homogeneous: Low-income countries of the Global South are more vulnerable^4,5^. People with high socioeconomic status, mostly populating the Global North, have been shown to have a larger-than-average impact on climate^6,7^. They directly impact climate through emissions largely related to energy generation and consumption. Simultaneously, this group impacts climate through investment decisions as well as role-model behavior, influencing the behavior and thus climate impact of larger population groups^8^. Previous research has shown that a large part of the effects of climate change can be explained by the emissions of a few major emitters, more precisely industrial carbon producers mostly established in the Global North^9^. In addition to this geographical injustice of climate change, large discrepancies between birth cohorts can be observed, not only regarding their greenhouse gas emissions but also their exposure to the effects of climate change^4^. Globally, calls for climate justice are emerging, and courts see an increase in so-called climate cases^10,11^. However, verdicts in these cases have often been impeded by the lack of robust evidence^12^. Judges have struggled to link greenhouse gas emission sources to the impacts of climate change on the plaintiffs^13^. Timely emission reduction and climate change mitigation can delay the onset of many climate change impacts, with benefits of climate action accumulating over decades and the first outcomes becoming visible well within one lifetime^14^.

The aim of this study is to directly relate per-capita GHG emissions to the global temperature increase experienced by individual birth cohorts over their lifetime, in different world regions, and for different scenarios (Shared Socioeconomic Pathways; SSPs)^15^. By quantifying the geographical and generational inequality of climate change, we bridge the gap between emission scenarios, temperature projections, and climate change impact. We calculate an index of the ratio of GHG emissions to experienced global warming, to quantify inequality on a standardized scale.

## Results and interpretation

We combine historical simulations and future projections of global mean surface temperature, historical and future GHG emission estimates, and population and life expectancy data. From these, we calculate per-capita emissions per lifetime and global temperature increase experienced over the lifetime by individual birth cohorts born between 1960 and 2018. The analyses are resolved by five world regions and different future emission scenarios represented by selected Shared Socioeconomic Pathways (SSPs). The experienced global warming over lifetime (ΔT) is the difference between the global mean surface temperature at the year of birth (YOB) and the global mean surface temperature at the year of death (YOD). We calculated ΔT for each birth cohort based on the global and regional average life expectancy. The lifetime per-capita greenhouse gas emissions (ΣGHG, read: lifetime emissions) are expressed as the amount of equivalent CO_2_ (i.e., the combined effect of the GHG equivalent to the amount of CO_2_ which would lead to the same greenhouse forcing), calculated for individual birth cohorts globally and regionally.

**Figure 1:**
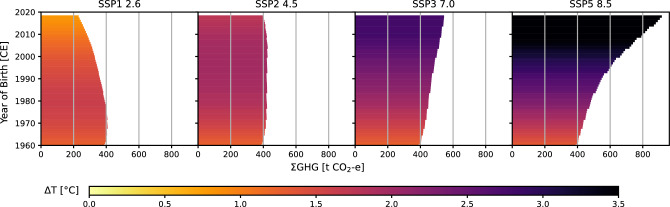
Experienced global warming (ΔT) and lifetime greenhouse gas emissions per-capita (ΣGHG) of people born between 1960 and 2018. The color of the bars indicates the magnitude of the global temperature increase (in °C) experienced by each birth cohort over their average lifetime. The length of the bars indicates the per-capita greenhouse gas emissions (in t CO_2_ equivalent), emitted globally during the average lifetime of each birth cohort. The panels show temperature and emission projections based on the Shared Socioeconomic Pathways (SSP) 1, 2, 3, and 5, respectively.

The global trends of ΣGHG and ΔT vary greatly between SSPs and birth cohorts (Fig. 1). Both SSP1 and SSP2 show turning points of decreasing ΣGHG and, with a time lag of several birth cohorts, decreasing ΔT. Both turning points occur much earlier in SSP1 compared to SSP2. Birth cohorts born after the respective turning points of ΔT will experience lesser amounts of warming over their lifetime, however, no birth cohort included in this study experiences a decrease in global mean surface temperature (negative ΔT) over their lifetime (the least affected cohorts experience ~ 0.8 °C increase). Both ΣGHG and ΔT continuously increase in SSP3 and SSP5, with the largest increase of both observed in SSP5.

Our analyses reveal that cohorts born after YOB 1980 experience a difference in ΔT of > 0.5 °C depending on the SSP. This equals 60% of the current global population. While the 2018 birth cohort experiences a global mean surface temperature of 2.05 °C above preindustrial in SSP1 at the end of their life, the same birth cohort experiences a temperature of 4.94 °C above preindustrial at YOD in SSP5 (Fig. S9, S10). These findings highlight the importance of emission reduction and the clear effect this will have on experienced global warming and related impacts.

**Figure 2:**
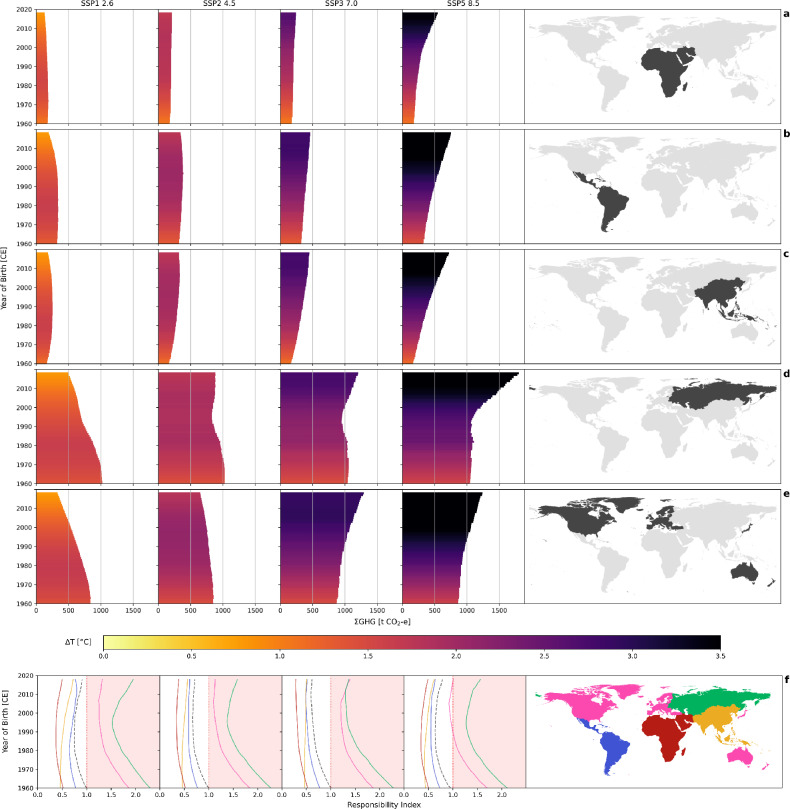
Regional patterns in experienced global warming (ΔT) and lifetime greenhouse gas emissions per-capita (ΣGHG) of people born between 1960 and 2018, and the Responsibility Index. Rows (**a**–**e**): The color scale indicates the magnitude of the global warming (in °C) experienced by each birth cohort over the regional average lifetime. The length of the bars indicates the per-capita greenhouse gas emissions (in t CO_2_ equivalent), emitted regionally during the average lifetime of each birth cohort. The columns show temperature and emission projections based on the Shared Socioeconomic Pathways (SSP) 1, 2, 3, and 5, respectively. The rows show the five world regions (defined according to SSP Database, 2012–2018, Available at: https://tntcat.iiasa.ac.at/SspDb) : (**a**) Middle East and Africa (MAF), (**b**) Latin America and the Caribbean (LAM), (**c**) Asia excluding OECD90, Middle East and REF countries (ASIA), (**d**) the former Soviet Union (REF), and (**e**) the OECD countries (OECD). Row (**f**) shows the Responsibility Index: Lifetime greenhouse gas emissions per-capita divided by the experienced global warming (ΣGHG/ΔT). The index is normalized to the emission/warming ratio of the global values for people born in 1960 assuming the SSP1 pathway. The area shaded in red highlights index values above this global 1960 value for SSP1.

The evaluation of ΣGHG for the individual world regions reveals distinct regional differences: MAF, LAM, and ASIA present lower ΣGHG across all birth cohorts and SSPs compared to REF and OECD, suggesting a grouping of the world regions into high-emission regions (REF and OECD, Fig. 2d-e) and low-emission regions (MAF, LAM, and ASIA) (Fig. 2a-c). All world regions exhibit similar trends of ΣGHG within one SSP, but differences are observed regarding the timing of turning points and magnitude of ΣGHG and ΔT. Note that the world regions differ in ΣGHG as of the first birth cohort investigated (YOB 1960), ranging from 160.7 t CO_2_-e (ASIA, SSP1 2.6, Fig. 2c) to 1040.5 t CO_2_-e (REF, SSP5 8.5, Fig. 2d). This difference further increases in SSP3 and SSP5, indicating an increase in the geographical inequality of GHG emissions in these SSPs. REF shows a temporary decrease of ΣGHG across all SSPs for the birth cohorts between YOB 1980–1995, caused by a decrease in life expectancy from YOB 1980 to YOB 2000 (Fig. S3). None of the investigated SSPs reaches a state of equally balanced ΣGHG between the world regions, i.e., with similar per-capita emissions.

The Responsibility Index is the ratio of ΣGHG divided by ΔT standardized to the YOB 1960 global average value, calculated for each birth cohort, world region, and SSP (Fig. 2f, Fig. S7). By definition, a value of one in the index represents the YOB 1960 status quo, all developments in the index can be interpreted relative to this baseline. The Responsibility Index is designed to quantify the responsibility of world regions (or individual nations) for having contributed to climate change in the past, but also to determine the obligation of future generations to reduce GHG emissions and mitigate climate change consequences they are confronted with because of past actions or inactions of nations. In this sense, the index can be understood to resemble a cost–benefit calculation, where the temperature increase represents the cost, while GHG emissions represent the benefit through the related economic developement. By calculating the ratio of ΣGHG to ΔT, we can comprehensively display the relationship between the two trends and allow for easier comparison of generations and SSPs. The mechanisms behind the index value can vary greatly between world regions as well as within birth cohorts. Drivers of the underlying factors (ΣGHG and ΔT) are of a complex nature and a variety of effects interfere with the climate system and, through the global average temperatures considered by the index, with the index values. Such effects include for example delayed emission feedbacks or globally inhomogeneous distributed rise in temperatures that cannot be accounted for within this study.

Across all SSPs and birth cohorts, the same grouping into high- and low-emission regions (corresponding to Global North and Global South, respectively) described earlier is observed, with the index of the high-emission regions remaining above the global average, and the index of the low-emission regions always remaining below it. Additionally, the global Responsibility Index remains below a value of one across all scenarios and birth cohorts born after 1960, meaning that the responsibility (the ratio of ΣGHG and ΔT) is higher for all generations born after the 1960 cohort. The individual world regions’ trends appear similar across the four SSPs, however, they are driven by different mechanisms. Across all SSPs, the index is rather high for the earliest birth cohorts, especially for the high-emission regions, with a decreasing trend for following birth cohorts. A decline of the index does not necessarily indicate lower GHG emission contribution (or responsibility) of regions, it rather expresses a change in the ratio of the underlying variables. For the given example, the trend is driven by stagnating (SSP3-5) ΣGHG paired with increasing ΔT (Fig. S7, S8).

For successive birth cohorts, the Responsibility Index begins to diverge between the SSPs. in SSP1 and SSP2, around YOB 1980, the decreasing trend begins to decelerate, stagnates, then turns into an accelerated increase around YOB 1990. This trend is caused by stagnating (SSP1) or slightly increasing (SSP2) ΔT paired with decreasing ΣGHG. The corresponding birth cohorts in SSP3 and SSP5 experience stagnating or slightly increasing ΣGHG paired with increasing ΔT, with the increase of ΔT being fastest in SSP5. The turning points observed here must not be seen as an indicator of declining responsibility or decreasing contribution of regions to climate change. It rather expresses the transition from the former, active responsibility of early birth cohorts to a passive and burden-laden obligation to maintain a habitable environment.

The birth cohorts born after 1990–2000 (depending on the SSP) experience an increase or stagnation in the Responsibility Index. This time, the index is driven by ΔT and ΣGHG decreasing, resulting in an increasing overall index. In SSP3, the corresponding birth cohorts experience a stagnating index caused by increasing ΣGHG and simultaneously increasing ΔT, with the ratio of the two roughly equal, causing stagnation of the index values. In SSP5, the same birth cohorts experience a distinct increase in the Responsibility Index, however, driven by different mechanisms compared to SSP1: Here, an extremely accelerated increase of both ΣGHG and ΔT drives the trend. Because ΣGHG increases faster in relation to ΔT, the index increases. The described trends show a time lag of a few years but can be recognized for all world regions comparably.

## Discussion

We use global mean surface temperatures derived from the SSPs as the only proxy of climate change. Despite the temperature being key to a number of climate change impacts, we neglect spatial heterogeneity of global warming, the regionally different starting points of warming as well as the mechanisms related to tipping points of the climate system^1,5^. Weather extremes are highly dependent on the absolute temperature increase as predicted by the SSPs and have been proven to affect generations unequally^4^. The frequency and impact of weather extremes such as the number of heatwave days and annual maximum one-day precipitation co-vary with global mean temperatures^16^. We expect this to additionally increase generational as well as geographical inequality, seeing that the effects of rising temperatures are not spatially homogeneous^1,5,16^. Furthermore, we focus on per-capita lifetime emissions only, without considering the historical inequality of emissions as revealed through cumulative emissions. Thereby, we can observe more precisely the impact individual birth cohorts have compared to each other and between world regions, in relation to the relative amount of warming they experience. For all trends and developments we describe, it must be noted that here, we refer to warming over a lifetime, neglecting the underlying absolute warming (compared to preindustrial) experienced by birth cohorts. The later a birth cohort is born, the higher the initial level of warming that the lifetime temperature increase adds to. Incorporating the temperature compared to preindustrial experienced by birth cohorts notably emphasizes inequality between birth cohorts (Fig. S6, S9, S10).

The index presented in this study depends on two variables. ΣGHG affects the index linearly, while ΔT has a non-linear effect. As a result, extreme experienced temperature increases do not proportionally reduce the index value, this mostly comes to effect in the high-emission scenarios. Compared to its variance, the extent of the temperature increase in the evaluation period still ensures a rather stable effect on the index. To facilitate comprehensibility of the index the simple relationship between the variables is assumed to represent responsibility sufficiently to evaluate trends in the data while maintaining a focus on the emissions. The mathematical relationship of the variables must however be noted, and the index is interpreted with adequate caution, especially where ΔT reaches extreme values.

The data used for this study do not cover socio-economic variability and its relation to GHG emissions on a spatial scale smaller than the world regions. Thus, intra-world region differences are neglected, as well as variability within the population of an individual country. The world region of Africa includes the countries with the highest and lowest per-capita emissions simultaneously, exemplifying the need for improvement of spatial resolution or a different parameter of grouping countries (i.e., by per-capita emissions or GDP). The data used for this study do not cover intra-national socio-economic variability and its relation to GHG emissions. Significant differences exist between nations and socio-economic groups within nations for example regarding the income and individual emissions. In the EU alone, differences of a factor of 20 were found upon the investigation of household GHG emissions, where high emissions were found to correlate with high income^17^.

Emission-free technologies of power generation have been in use for centuries globally (water- and wind energy). The advancement of such technologies to an industrial-level efficiency and extent has only been possible due to high emissions throughout the process. Thus, initially higher GHG emissions of a generation (causing temperature increase and adding to the responsibility of the generation for future climate change) should arguably remedy some of the responsibility. In contrast, not exhausting such emission-free technologies despite the availability of financial, technological, and material resources could be understood as additional responsibility for the effects of climate change. Such political, technological, and socio-economic circumstances vary greatly across world regions and can change rapidly with major events happening, such as the onset of a pandemic or a war.

Despite these simplifications, our study clearly demonstrates the extensive geographical and generational inequalities of climate change and proposes a methodological approach to quantify these. Additionally, our approach can be applied to datasets of any temporal or spatial resolution, given that such data are available, promising a variety of future applications and additional results when applied to different input datasets. The attribution of GHG emissions to an individual state is rarely straightforward, just as much as the question of who has a right to emissions, historical responsibility, and future emissions budgets^18^. However, Ott et al.^17^ provide convincing arguments that it is possible to assign emissions and responsibility at a country-scale, and even with regard to single extreme events^19^.

Our analyses reveal an inequality of ΣGHG and ΔT between individual birth cohorts and regions, most prominently a clear distinction between high- and low emission regions. Arguably among the earliest and most prominent approaches to quantify generational inequality is the Generational Environmental Debt as described by Azar and Holmberg. Despite the common basis of generational justice, their study uses a much more economy-centered approach, whereas we focus on the real-lifetime emissions and experienced warming and thereby choose a less economic, more individuum-centered method^20^. Such economy-centered approaches have been refined lately, yielding results that match those presented here^21,22^. Van Houtan et al.^23^ have presented a method closely related to the one presented here. They introduce an index of local climate disparity, based on emissions and local warming. While achieving a higher spatial resolution, their approach omits the generational focus that we introduce. Generally, the results presented by Van Houtan et al. and ours are in very good agreement, especially with regard to the spatial inequality of emissions where an overwhelming majority of emissions originate from nations of the Global North^23^. Previous research has shown that the highest emitting countries are also the ones least vulnerable to the effects of climate change, an aspect that amplifies geographical inequality that is not accounted for in this study^24^.

The emissions caused by one birth cohort only unfold their impact on the climate with a certain time lag. The birth cohorts do not only experience the consequences of their own lifetime emissions, but of those that previous generations emitted. Hypothetically, a future birth cohort might be born without having emitted a single gram of CO_2_ but will continue to suffer from global warming caused by emissions attributed to past birth cohorts. Simultaneously, the obligation to lower emissions is subject to the same time lag in emissions. Despite suffering from increasing ΔT, birth cohorts must lower their ΣGHG so that later birth cohorts can experience decreasing ΔT. At the same time, each birth cohort determines the burden of future cohorts through their emissions as a cause of future temperature increase, irrespective of the consequences (ΔT) they are faced with themselves. Within this observation lies the central problem of generational inequality: There will be birth cohorts suffering from the effects of high ΣGHG of previous birth cohorts while carrying the burden of having to reduce their lifetime emissions to allow future birth cohorts to experience decreasing amounts of ΔT. The causes (high emissions causing temperature increase) as well as the effects (higher temperatures, the obligation to lower emissions) of climate responsibility extend across lifetimes and generations, impeding precise quantification and the attribution to individual birth cohorts. Successive birth cohorts either face or escape the consequences or the climate (ir-)responsible behavior of previous generations. Simultaneously, and irrespective of the consequences (ΔT) confronted with, each birth cohort determines the consequential burden future cohorts are faced with through their emissions as a cause of further temperature increase.

The implications deriving from our results can hardly be interpreted further without agreeing on an underlying concept of justice, raising the moral question if all people should have an equal right to emissions (distributive justice) and if that applies to all generations^25^? Finding an answer to the question of justice and responsibility is outside the aim and scope of this study and has been touched upon elsewhere, however, must be kept in mind when working with the implications of our results. In a wider sense, our results raise ethical and moral questions. Who is responsible for which emissions, how strongly should cumulative historical emissions be weighted, and who is obliged to reduce GHG emissions in the future? Can birth cohorts be held accountable for the GHG emissions caused while still underaged? We provide valuable context and quantitative results to support the debate.

The current generations are not only the first to feel the effects of anthropogenic climate change^5^. We show that they are also the first ones having a realistic chance of seeing decreasing amounts of warming compared to preceding generations^26^, given the commitment to a low-emission future in a global effort.

## Methods

**Figure 3:**
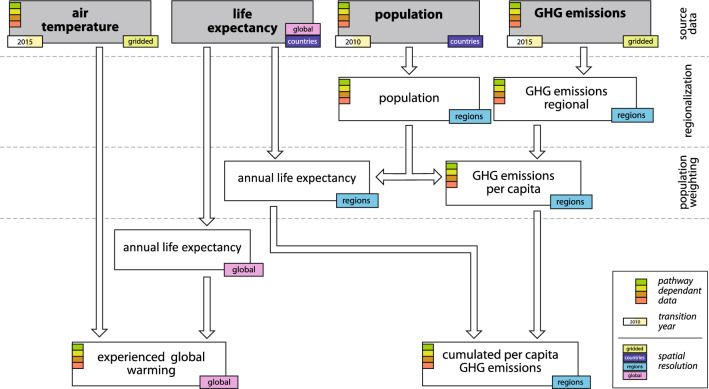
Workflow of the data analysis. We combine historical simulations and future projections of near-surface (2 m) air temperature^27^, historical data and future scenarios of greenhouse gas emissions^29^, past and future population data^30,31^, and life expectancy data^7^ to calculate (1) the experienced global warming during a lifetime and (2) the lifetime greenhouse gas emissions per-capita of people born between 1960 and 2018. The source data sets are listed in the gray boxes, the processed data sets in the white boxes. Colors on the left side of the boxes indicate pathway-dependent data sets using the Shared Socioeconomic Pathways (SSP) 1, 2 ,3 and 5. The tags on the bottom right of the boxes indicate the spatial resolution of the data sets. The year in the bottom left insets state the transition year between historical and projected data in each source dataset if applicable.

This study calculates the experienced global warming during a lifetime and the lifetime greenhouse gas emissions per-capita for people born between 1960 and 2018. The workflow of the analyses is shown in Fig. 3. The data sets used (population, life expectancy, air temperature and greenhouse gas emissions) combine historical data (1960–2018) and future projections (2010–2100 for population and life expectancy, 2015–2100 for air temperature and greenhouse gas emissions). Projections are based on the Shared Socioeconomic Pathways SSP1 2.6, SSP2 4.5, SSP3 7.0 and SSP5 8.5^27,28^. The data sets were transformed if necessary to have an annual resolution and were aggregated globally as well as for the five world regions Middle East and Africa (MAF), Latin America and the Caribbean (LAM), Asia excluding OECD90, Middle East and REF countries (ASIA), the former Soviet Union (REF), and the OECD countries (OECD)^27,28^ (Fig. S1).

Annual country population data from 1960 to 2010^30^ was combined with decadal population projections to 2100 for the different SSP^31^, which were linearly interpolated. Annual global and regional populations were calculated from the country data (Fig. S2). Global and country-level life expectancy data^32^ with a 5-year temporal resolution was interpolated to annual values and rounded to the nearest integer. To account for the regional differences in child mortality, which have a strong influence on the life expectancy at birth, we used the life expectancy at the age of 5 for each birth cohort and added 5 years to obtain the total life expectancy for that cohort in their year of birth. Global and regional life expectancy estimates were calculated as the population-weighted average of the respective countries (Fig. S3).

Gridded data sets of anthropogenic greenhouse gas emissions were used to combine historical emissions with future estimates according to the different SSP from 1960–2100^33^. We selected the long-lived greenhouse gases CO_2_ and CH_4_, which are available as gridded data sets and together account for about 90% of the change in effective radiative forcing since the preindustrial era due to anthropogenic emissions^34^. Their quantities are expressed as t CO_2_ equivalent estimating a long-lived Global Warming Potential (GWP-100) for CH_4_ of 29.8 and summed^35^. Emissions from air traffic are omitted from this analysis because they cannot be attributed to specific regions. Anthropogenic aerosols, which have a negative radiative forcing, are also omitted, as they have an impact on human health and should not appear in this study as a compensation of greenhouse gas emissions^35^. Similarly, the radiative forcing from land use change has been omitted because it is not clear that a negative forcing due to land use change has a positive effect on the environment overall. From the gridded emission data sets, the total annual emissions were calculated globally and regionally (Fig. S4), and the annual per-capita emissions were calculated globally and regionally dividing the total emissions by the annual global or regional population (Fig. S5). The annual global mean near-surface (2 m) temperature was calculated from gridded data for the different SSP (Fig. S6)^27^.

For each birth cohort, the experienced global warming during a lifetime (ΔT) is then calculated as the global mean air temperature at the end of the life (based on the global and regional average life expectancy at the age of 5) minus the global mean temperature at birth (Figs. 1 and 2, S7). The lifetime greenhouse gas emissions per-capita (ΣGHG) are calculated for each birth cohort as the globally and regionally emitted GHG during the average global or regional lifetime (Figs. 1 and 2, S7). Figure 1 and Fig. 2 are combined representations of the global and regional ΔT and ΣGHG. A “Responsibility Index” is calculated for each birth cohort by standardizing the qotient ΣGHG/ΔT to the global baselie value of the borth cohort 1960 (Fig. 2, S7).

## Supplementary Information

Supplementary Figures.

## Data Availability

All datasets used for the analyses of this study are available from public repositories (see references in the Methods section).
