# Supplementary material for: Quantifying generational and geographical inequality of climate change
Source: Sci Rep. 2023 May 25;13:8483. doi: 10.1038/s41598-023-35690-8 (PMC10212934; doi:10.1038/s41598-023-35690-8)
Supplement: Supplementary file 1 — Supplementary Figures. [file 41598_2023_35690_MOESM1_ESM.pdf]

Supplementary Material

Quantifying generational and geographical inequality of climate change

Emma Hadré[1*+], Jonas Küpper[1+], Janina Tschirschwitz[1+], Melissa Mengert[1], Inga Labuhn[1]

[1]University of Bremen, Institute of Geography, Celsiusstr. 2, 28359 Bremen, Germany

[+]These authors contributed equally to this study.

*Corresponding Author

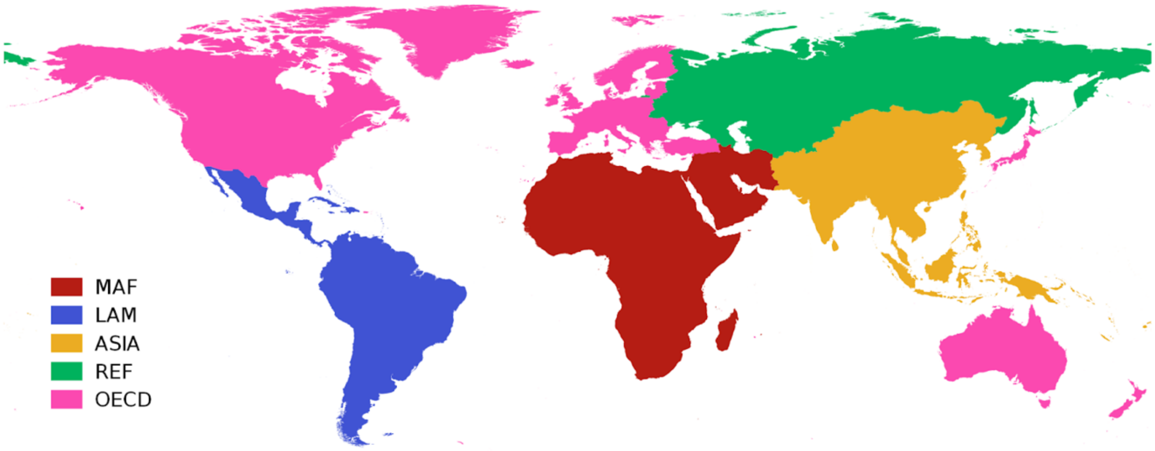

Figure S1: The five world regions used to evaluate geographical differences in regional per-capita greenhouse gas emissions in relation to global warming*: Middle East and Africa (MAF), Latin America and the Caribbean (LAM), Asia excluding OECD90, Middle East and REF countries (ASIA), the former Soviet Union (REF), and the OECD countries (OECD); defined according to the SSP Database[13].*

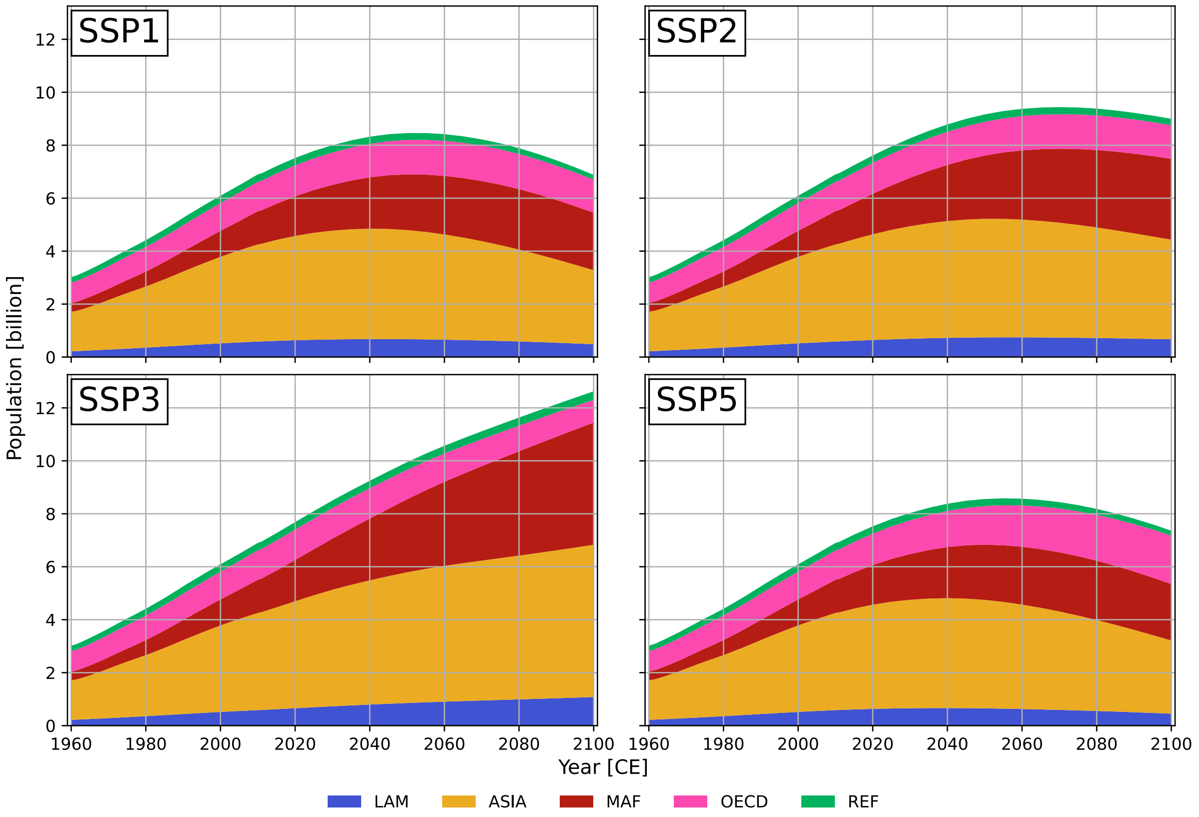

Figure S2: Global and regional population development from 1960 to 2100. *Historical population data[26] is combined with population projections[27] according to the Shared Socioeconomic Pathways (SSP) 1, 2, 3 and 5[32]. The five world regions are defined in Fig. S1. The sum of these five regions gives the global population.*

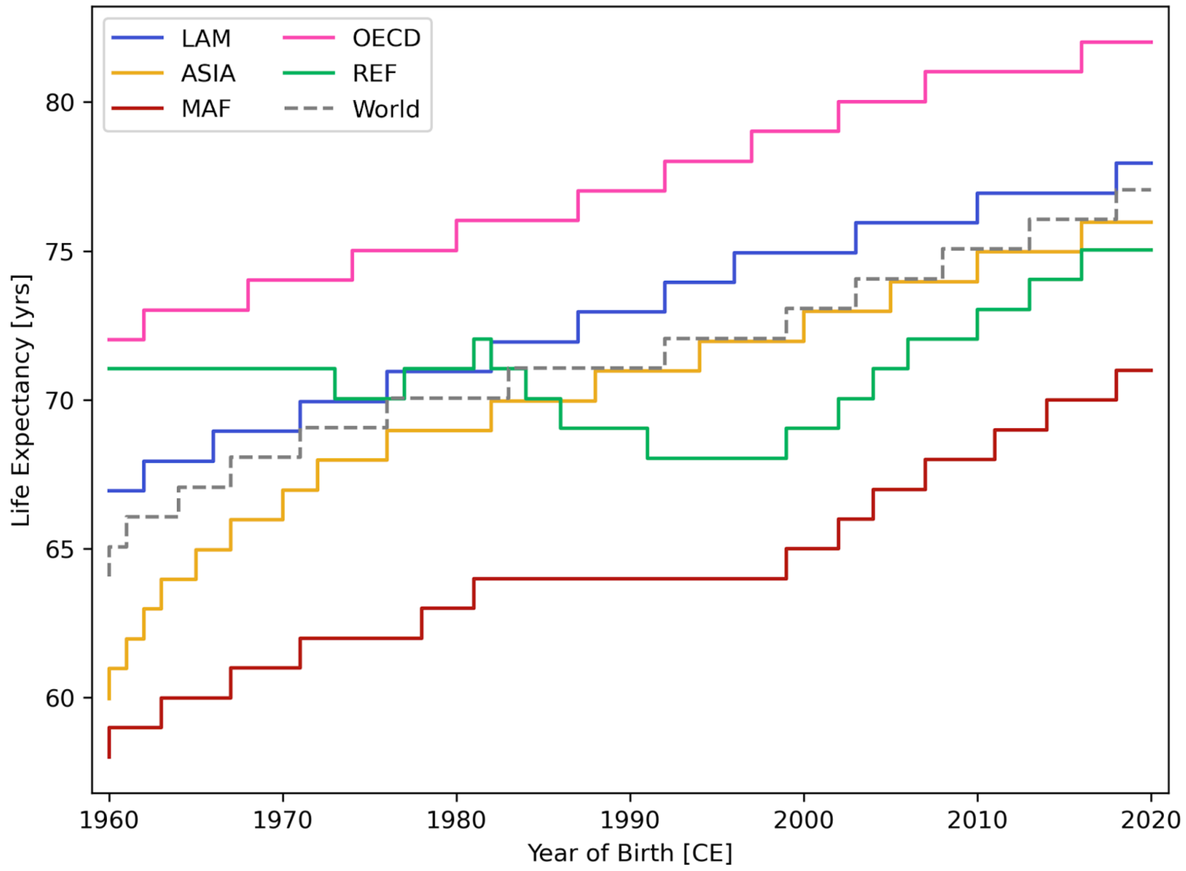

Figure S3: Global and regional population life expectancy from 1960 to 2018. *Country-level life expectancy data were obtained from The United Nations[28] with a 5-year temporal resolution, interpolated to annual values and rounded to the nearest integer. Shown is the life expectancy at the age of 5 for each birth cohort with 5 years added to obtain the total life expectancy for that cohort in their year of birth. Global and regional life expectancy estimates were calculated as the population-weighted average of the respective countries. The five world regions are defined in Fig. S1.*

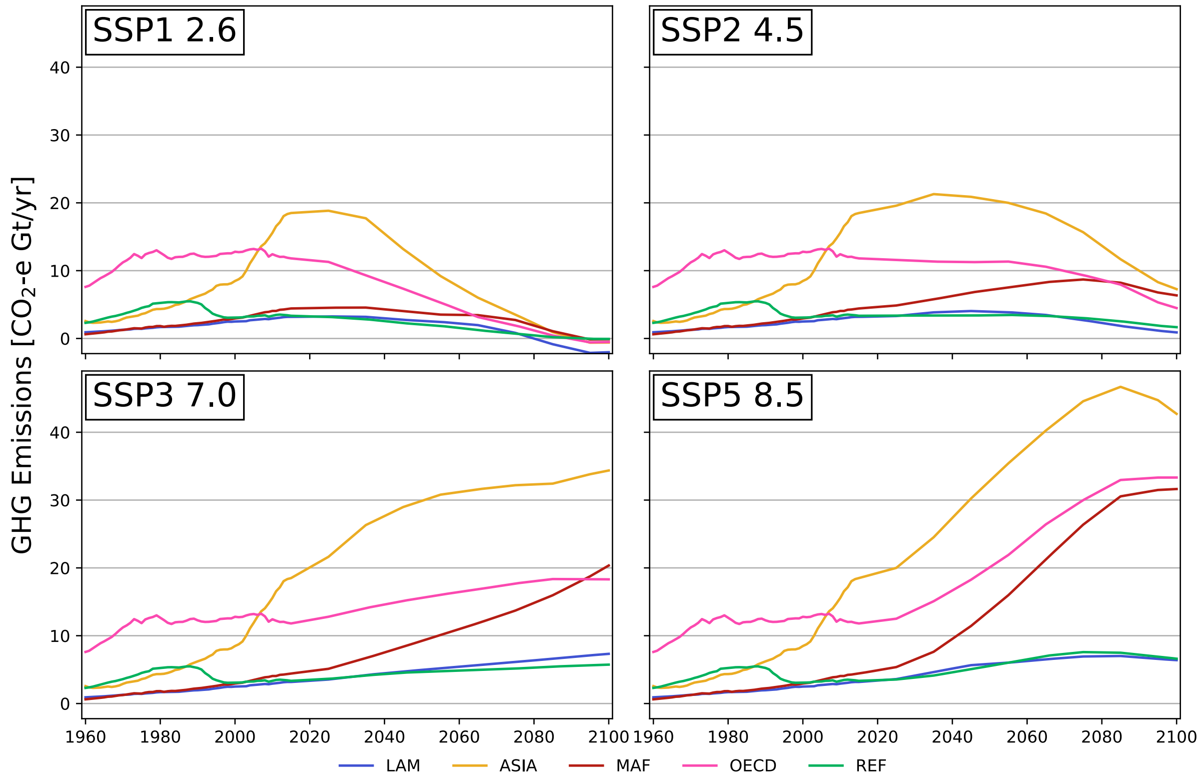

Figure S4: Annual regional greenhouse gas (GHG) emissions from 1960 to 2100. *Shown are sum of CO_2 and CH_4 emissions, expressed as CO_2 equivalent in Gt/year. Historical emissions and future projections for the Shared Socioeconomic Pathways (SSP) 1, 2, 3 and 5 are obtained from the SSP Database[13]. The five world regions are defined in Fig. S1.*

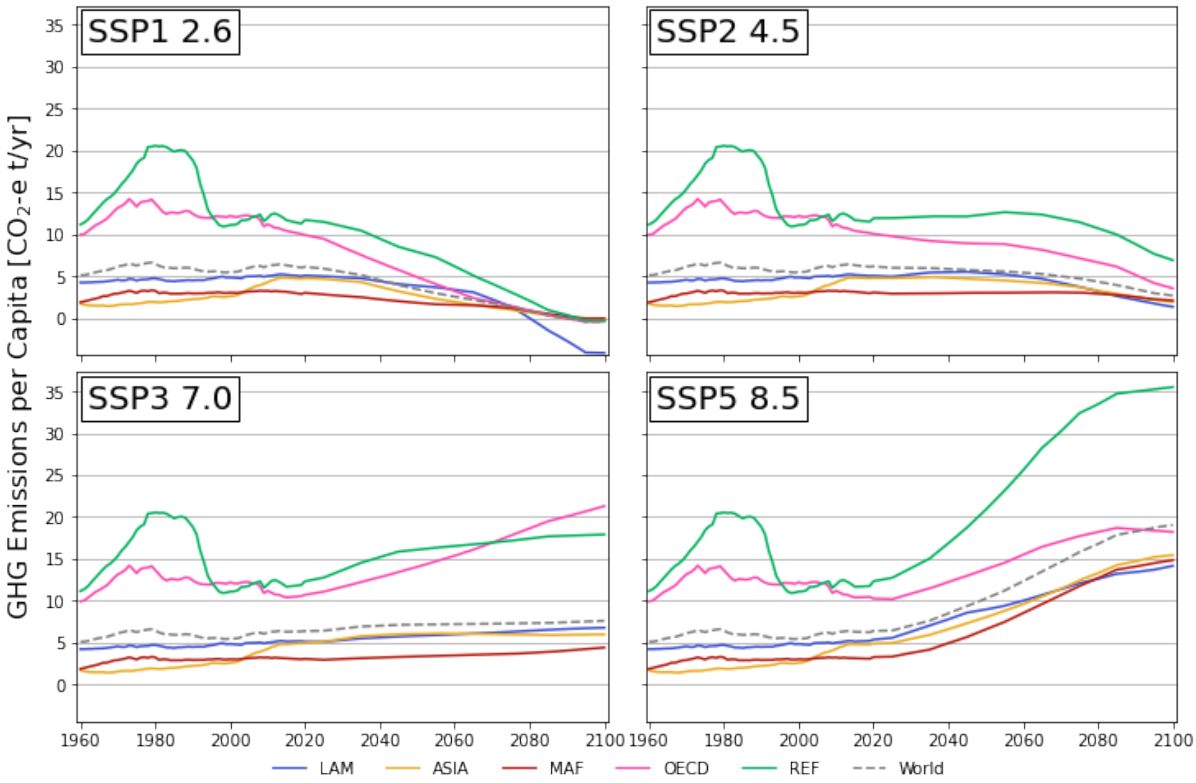

Figure S5: Annual global and regional per-capita greenhouse gas (GHG) emissions from 1960 to 2100. *Shown are CO_2 and CH_4 emissions expressed as CO_2 equivalent in Gt/year. Historical emissions and future projections for the Shared Socioeconomic Pathways (SSP) 1, 2, 3 and 5 are obtained from the SSP Database. The per-capita emissions are calculated from the total annual emissions (Fig. S4) divided by the annual global or regional population[26,27] (Fig. S2). The five world regions are defined in Fig. S1.*

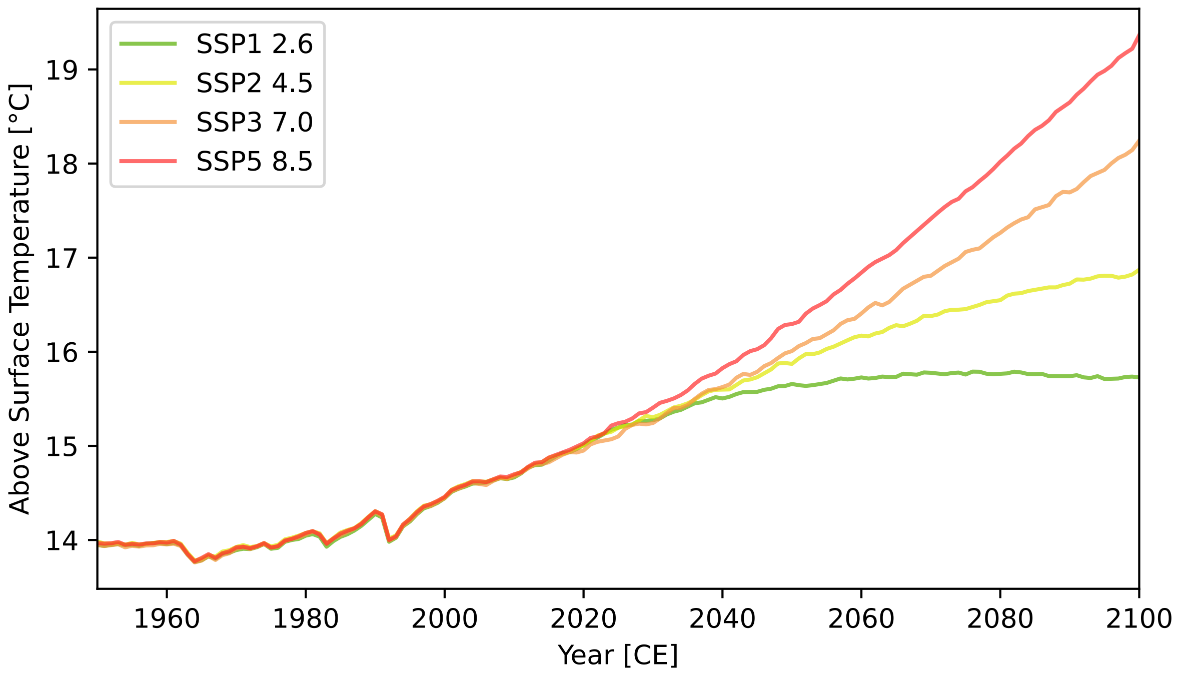

Figure S6: Global mean near-surface (2 m) air temperature from 1950 to 2100. *Shown is a multi-model mean of historical simulations and projections based on the Shared Socioeconomic Pathways (SSP) 1, 2, 3 and 5*[13]*. Gridded Data were retrieved from the KNMI/WMO website Climate Explorer (reference "KNMI Climate Explorer," n.d.) by selecting "monthly CMIP6 scenario runs" and "CMIP6 mean" with preprocessing to annual means. The global mean is weighted by grid cell area. CMIP6 models included are ACCESS-CM2, ACCESS-ESM1-5, AWI-CM-1-1-MR, BCC-CSM2-MR, CAMS-CSM1-0, CanESM5, CanESM5-CanOE, CESM2, CESM2-WACCM, CMCC-CM2-SR5, CNRM-CM6-1, CNRM-CM6-1-HR, CNRM-ESM2-1, EC-Earth3, EC-Earth3-Veg, FGOALS-f3-L, FGOALS-g3, FIO-ESM-2-0, GFDL-CM4, GFDL-ESM4, GISS-E2-1-G, HadGEM3-GC31-LL, HadGEM3-GC31-MM, INM-CM4-8, INM-CM5-0, IPSL-CM6A-LR, K-ACE-1-0-G, MCM-UA-1-0, MIROC6, MIROC-ES2L f2, MPI-ESM1-2-HR, MPI-ESM1-2-LR, MRI-ESM2-0, NESM3, NorESM2-LM, NorESM2-MM, UKESM1-0-LL (specific citations for aforementioned models see Tebaldi et al.(2021) and CIESM/ Huang (2019))*[24,33]*.*

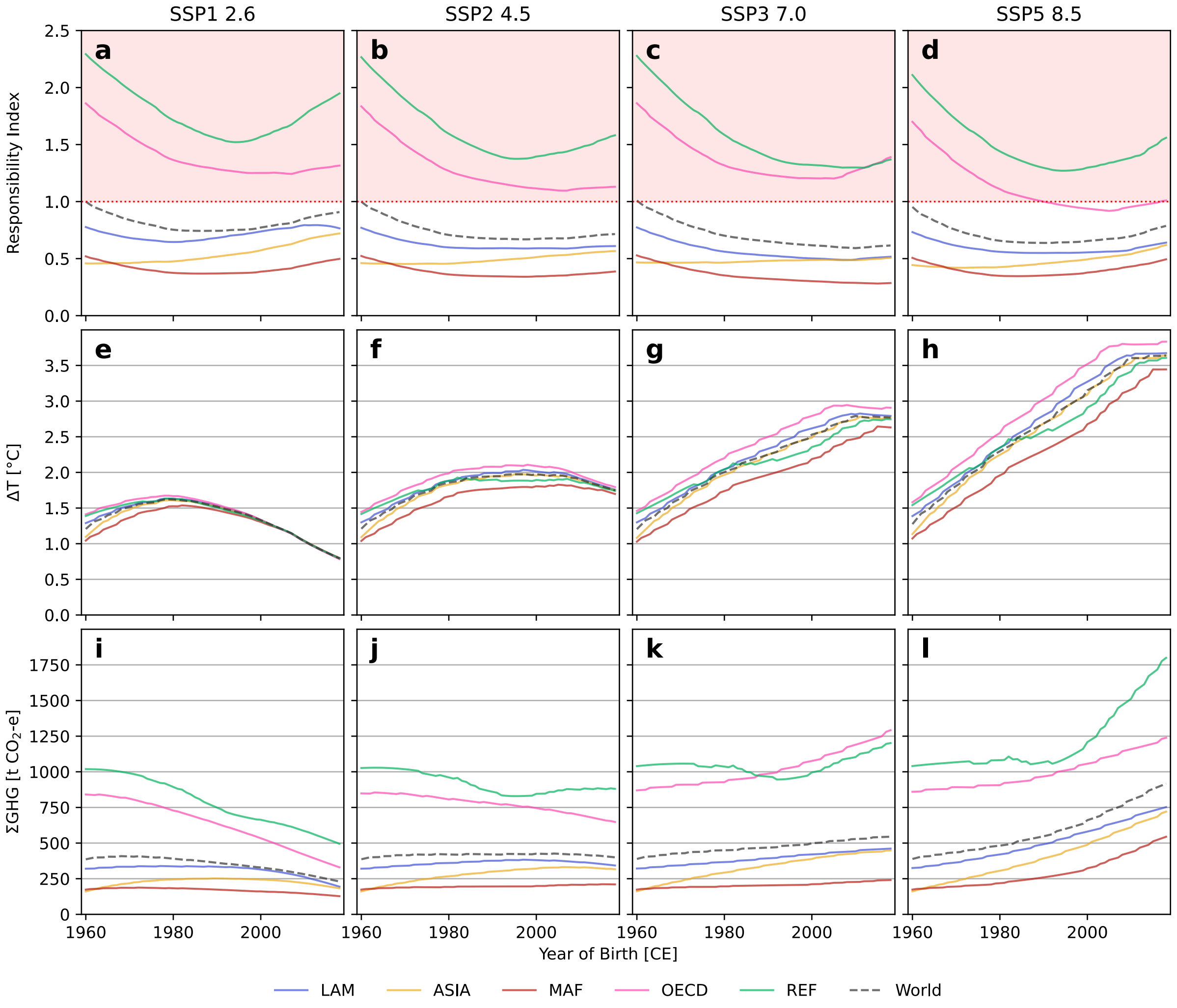

Figure S7: a-d) The "Responsibility Index". Lifetime greenhouse gas emissions per-capita divided by the experienced global warming (ΣGHG/ΔT) of people born between 1960 and 2018. *The index is normalized to the emission/warming ratio of the global values for people born in 1960 assuming the SSP1 pathway. The area shaded in red highlights index values above this global 1960 value for SSP1.* ***e-h) The experienced global warming of people born between 1960 and 2018 (ΔT)****, which represents the magnitude of the global temperature increase (in °C) experienced by each birth cohort over their average lifetime.* ***i-l) Lifetime greenhouse gas emissions per capita of people born between 1960 and 2018 (ΣGHG)****, which indicate the per-capita greenhouse gas emissions (in t CO_2 equivalent) emitted regionally during the average lifetime of each birth cohort.*

The different columns show the temperature and emission projections and the resulting index from the Shared Socioeconomic Pathways (SSP) 1, 2, 3, and 5, respectively. The dashed gray line displays the global data, the colored lines the regional data for the five world regions defined according to the SSP Database[13]: Middle East and Africa (MAF), Latin America and the Caribbean (LAM), Asia excluding OECD90, Middle East and REF countries (ASIA), the former Soviet Union (REF), and the OECD countries (OECD).

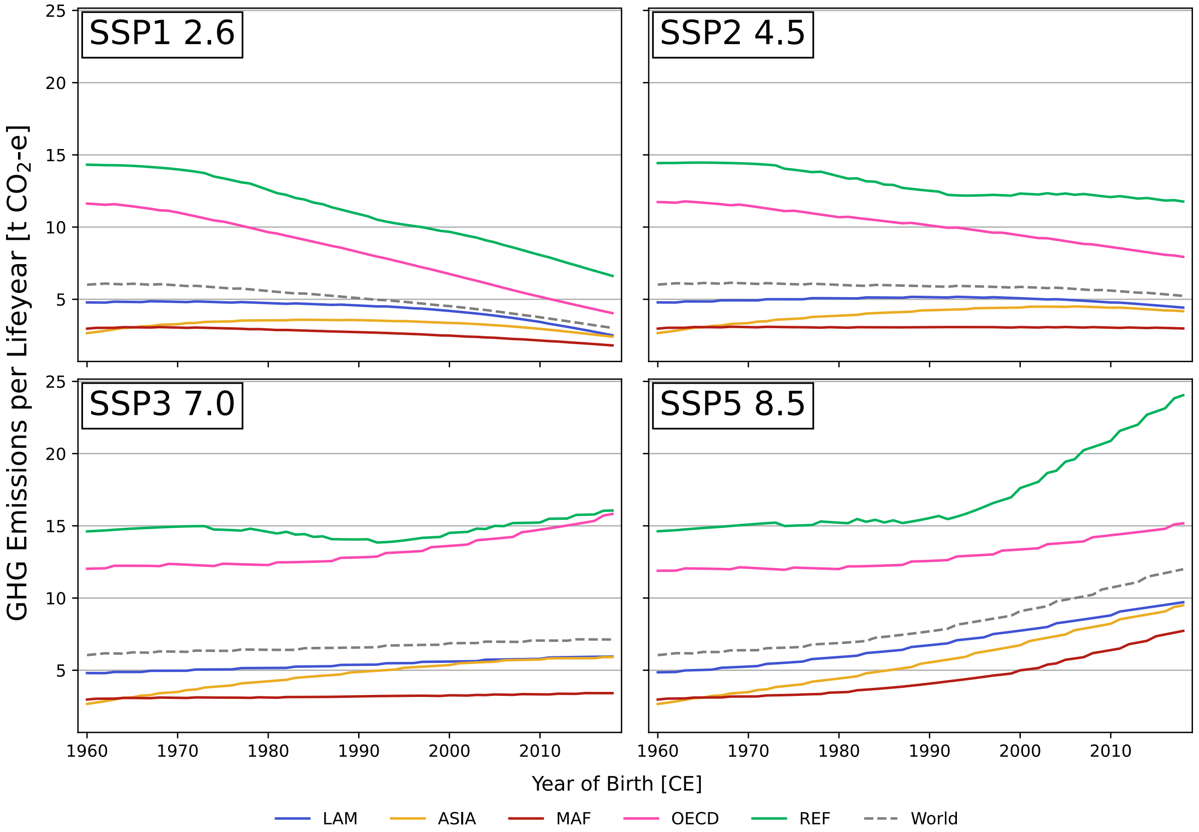

Figure S8: Lifetime greenhouse gas (GHG) emissions per-capita per year of life of people born between 1960 and 2018. *Note that this figure is related to Fig. S7i-l, but the lifetime GHG emissions per-capita are divided by the life expectancy at the age of 5 for each birth cohort. GHG emissions include CO_2 and CH_4, expressed as CO_2 equivalent (t), and are based on historical emissions and future scenarios according to the Shared Socioeconomic Pathways (SSP) 1, 2, 3 and 5*[34]*. The lifetime GHG emissions are calculated based on the global and regional total annual per-capita GHG emissions (Fig. S4, S5) and the average global and regional lifetime for each birth cohort. Life expectancy data are obtained from The United Nations*[28] *and are shown in Fig. S3. The five world regions are defined in Fig. S1.*

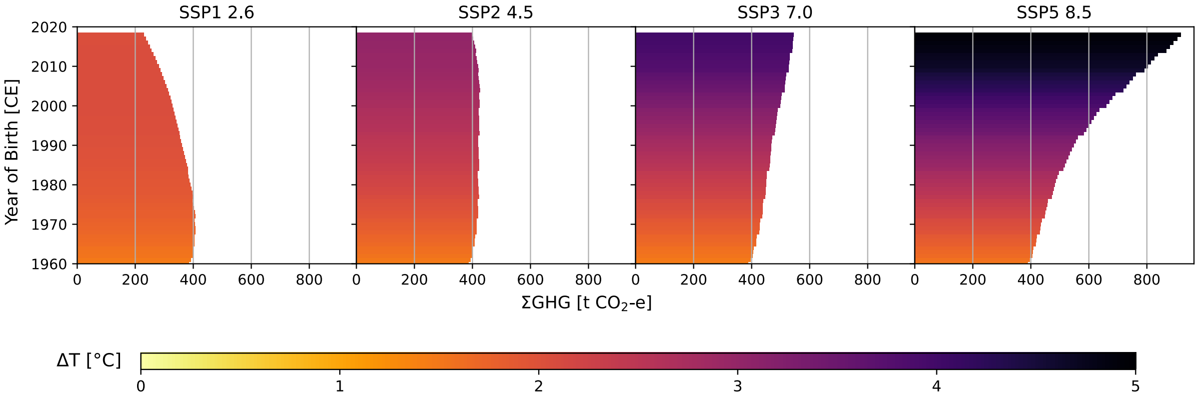

Figure S9: Experienced global warming and lifetime greenhouse gas emissions per-capita of people born between 1960 and 2018 (YOB) compared to pre-industrial temperatures. *The color of the bars indicates the magnitude of the global temperature increase (in °C) from a pre-industrial global temperature of 13.70 °C (1850–1880 average) to the projected temperature at the end of the average lifetime of each birth cohort. The length of the bars indicates the per-capita greenhouse gas emissions (in t CO_2 equivalent), emitted globally during the average lifetime of each birth cohort. The panels show*

temperature and emission projections based on the Shared Socioeconomic Pathways (SSP) 1, 2, 3, and 5, respectively[24,34]. Note that this figure is similar to Figure 1, but the global temperature increase experienced by each birth cohort over their lifetime is replaced by the global average temperature increase from pre-industrial time to the end of the lifetime of the respective cohort.

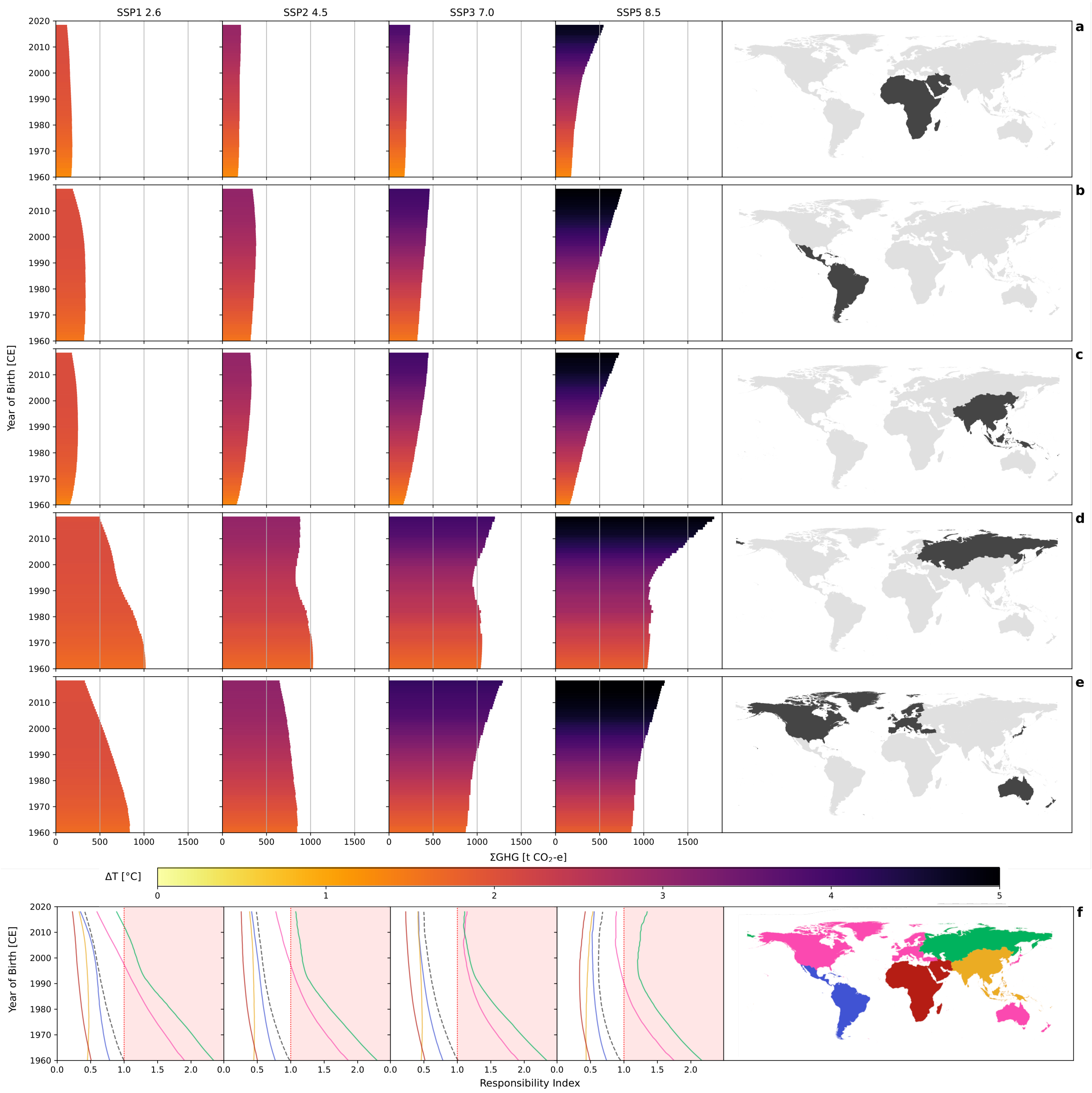

Figure S10: Regional patterns in experienced global warming and lifetime greenhouse gas emissions per-capita of people born between 1960 and 2018 compared to pre-industrial temperatures. *The color of the bars indicates the magnitude of the global temperature increase (in °C) from a pre-industrial global temperature of 13.70 °C (1850–1880 average) to the projected temperature at the end of the regional average lifetime of each birth cohort. The length of the bars indicates the per-capita greenhouse gas emissions (in t CO2 equivalent), emitted regionally during the average lifetime of each birth cohort. The columns show temperature and emission projections based on the Shared Socioeconomic Pathways (SSP) 1, 2, 3, and 5, respectively[24,34]. The rows show the five world regions defined in Figure S1. Note that this figure is related to Figure 2, but the global temperature increase experienced by each birth cohort over their lifetime is replaced by the global average temperature increase from pre-industrial time to the end of the lifetime of the respective cohort.*
